# Supplementary material for: Improving inpatient provider-initiated HIV testing and counseling in Sierra Leone
Source: PLoS One. 2020 Jul 24;15(7):e0236358. doi: 10.1371/journal.pone.0236358 (PMC7380619; doi:10.1371/journal.pone.0236358)
Supplement: Attachment — Submitted filename: Response to Reviewers.docx [file pone.0236358.s003.docx]

Dear Editor,

We responded to each point raised by the editor and reviewers. Our responses are on the second column.

| Please include the following items when submitting your revised manuscript: | RESPONSE |
| --- | --- |
| - A rebuttal letter that responds to each point raised by the academic editor and reviewer(s). This letter should be uploaded as separate file and labeled 'Response to Reviewers'. | √ |
| - A marked-up copy of your manuscript that highlights changes made to the original version. This file should be uploaded as separate file and labeled 'Revised Manuscript with Track Changes'. | √ |
| - An unmarked version of your revised paper without tracked changes. This file should be uploaded as separate file and labeled 'Manuscript'. | √ |
| Please note while forming your response, if your article is accepted, you may have the opportunity to make the peer review history publicly available. The record will include editor decision letters (with reviews) and your responses to reviewer comments. If eligible, we will contact you to opt in or out. |  |
| We look forward to receiving your revised manuscript. Kind regards, Olanrewaju Oladimeji, MB;BS, Ph.D. Academic Editor PLOS ONE  **Journal Requirements:** |  |
| When submitting your revision, we need you to address these additional requirements. |  |
| 1. Please ensure that your manuscript meets PLOS ONE's style requirements, including those for file naming. The PLOS ONE style templates can be found at |  |
| https://journals.plos.org/plosone/s/file?id=wjVg/PLOSOne_formatting_sample_main_body.pdf and |  |
| https://journals.plos.org/plosone/s/file?id=ba62/PLOSOne_formatting_sample_title_authors_affiliations.pdf |  |
|  |  |
| 2. Please address the following: |  |
| a) Please include additional information regarding the survey or questionnaire used in the study and ensure that you have provided sufficient details that others could replicate the analyses. For instance, if you developed a questionnaire as part of this study and it is not under a copyright more restrictive than CC-BY, please include a copy, in both the original language and English, as Supporting Information. | A table with list of QI indicators used for collecting data attached. |
| b) Please ensure you have thoroughly discussed any potential limitations of this study within the Discussion section. Additional Editor Comments (if provided): | Potential limitations are included. |
| There is a need to be very clear on the design of the study | We added relevant detail to the methods section to emphasize that this is a non-experimental design that reports time-series data. |
| Figure of a PDSA cycle will be helpful to understand and better appreciate the QI within the short period | Added to methods section |
| Reference 1 is missing in the text | Added |
| Please include the IRB approval numbers | The project received non-research determination from the Columbia University IRB, protocol AAAR7670. The CDC CGH HSR tracking # is 2019-051. |
| Need to cite relevant supporting findings | Added references especially in discussion section |
| Authors should use the COREQ checklist or other relevant checklists listed by the Equator Network such as the SRQR to ensure complete reporting |  |
| Reviewers' comments: |  |
| Reviewer's Responses to Questions  **Comments to the Author**  1. Is the manuscript technically sound, and do the data support the conclusions? The manuscript must describe a technically sound piece of scientific research with data that supports the conclusions. Experiments must have been conducted rigorously, with appropriate controls, replication, and sample sizes. The conclusions must be drawn appropriately based on the data presented. Reviewer #1: Yes Reviewer #2: Partly Reviewer #3: Yes |  |
| 2. Has the statistical analysis been performed appropriately and rigorously?  Reviewer #1: Yes Reviewer #2: No Reviewer #3: Yes |  |
| 3. Have the authors made all data underlying the findings in their manuscript fully available? The [PLOS Data policy](http://www.plosone.org/static/policies.action#sharing) requires authors to make all data underlying the findings described in their manuscript fully available without restriction, with rare exception (please refer to the Data Availability Statement in the manuscript PDF file). The data should be provided as part of the manuscript or its supporting information, or deposited to a public repository. For example, in addition to summary statistics, the data points behind means, medians and variance measures should be available. If there are restrictions on publicly sharing data—e.g. participant privacy or use of data from a third party—those must be specified. Reviewer #1: Yes Reviewer #2: Yes Reviewer #3: Yes |  |
| 4. Is the manuscript presented in an intelligible fashion and written in standard English? PLOS ONE does not copyedit accepted manuscripts, so the language in submitted articles must be clear, correct, and unambiguous. Any typographical or grammatical errors should be corrected at revision, so please note any specific errors here. Reviewer #1: Yes Reviewer #2: Yes Reviewer #3: Yes |  |
| **5. Review Comments to the Author** Please use the space provided to explain your answers to the questions above. You may also include additional comments for the author, including concerns about dual publication, research ethics, or publication ethics. (Please upload your review as an attachment if it exceeds 20,000 characters) |  |
| **Reviewer #1:**  **Abstract** | **RESPONE** |
| 1. It will be better in the authors included a sentence on PITC as the opening statement of the abstract. To enhance the comprehensibility of the concept from the abstract as it is in the introduction. | We have edited the abstract for clarity. |
| 2. It will be important for the authors to state clearly the objective(s) of this study. In as much as it was aimed at achieving quality improvement collaboration. The exact objective(s) of the study has not been clearly brought to light in the background/introduction. | We have added this to the abstract. |
| 3. It is also important for the authors to state clearly the study design used. It appears quasi experimental study/an interventional study with a before and after design. However, it has not been succinctly stated. | This is correct, the QIC used time series data to describe change over time; we added detail to the methods section to highlight this point. |
| Introduction |  |
| 1. Reference on line 49 needs to be revised | Revised. |
| Methods 1. The authors have provided a detailed and elaborate narration on the QIC plan and design. However, since this was a study conducted in 10 hospitals, details of actual QIC plan and design were sketchy e.g. the composition of each facility team, the numbers of quarterly held, the number of monthly site support and QI coaching visits received in the methods section as against the results section etc. | The team composition (HCWs from wards and counselors from HIV clinics), # of learning sessions and supportive supervisions is described and moved to method section. |
| Project implementation 1. It is also important to be clear about the designation of the head of each facility team as well as if the leaning sessions were conducted in facility or elsewhere. Also, the authors need to provide information on measures put in place to ensure similarity and validity of the learning sessions contents if provided by different group of people. | Team structure varied from hospital to hospital.  Learning sessions were conducted out of the facility, bringing the 10 hospital teams together; all participants thus experienced identical training. |
| Results 1. The authors should consider including the interquartile range of the median pre and post test scores of the participants for completeness sake. Furthermore, it is suggested that the authors carry out a statistical test of difference of median scores (pre and post test) e.g wilcoxon signed rank test so as to have statistical evidence of improvement in the test scores following the training. | Paired t test used and revised in the result section. |
| 2. The authors should also consider providing information on demographic characteristic of the participants e.g. age, gender, highest education qualification, duration of practice and previous attendance of such training etc if the information were collected in the course of the study. This will go along way in enhancing the readers' understanding of the participants and as well as promoting reproducibility of QIC in other similar settings | This data was not collected. |
| 3.Stock out of kits was reported by 6 of the 10 facilities but the authors did not mention any measures instituted to mitigate against stock out at the commencement of the study as well as the likely effects of the stock out on the final outcome for example in those facilities where stock out was experienced, what proportion of those inpatients who met the inclusion criterion was missed due to stock out etc. | Added few description on result and discussion section to clarify that testing kit stock outs experienced, but had no impact on the HIV testing coverage during the project period as HCWs engaged leadership and replenished the supply. But, highlighted the challenge as it would be a barrier for maintaining PITC services and HIV testing coverage. |
| Discussion 1.The authors may need to revise the discussion section making it more robust and exhaustive bringing to light the implications of the findings of the study in the light of other similar studies. Furthermore, it is important that the authors also bring to context how the findings of the study will impact practice in this setting and other and other settings alike. | Revised the discussion considering other studies, and highlighted the significance of the findings. |
| References 1.It is important that the authors revise the references in line with the specified journal style e.g reference 6 | Revised. |
| Reviewer #2:  Overall it is well written and straight forward. The following limitations are noted: a) The research design could have been improved by using a stepped wedge cluster randomised controlled trial by having half of the hospitals start in the experimental condition while the rest would receive not intervention as control groups. The control group should have then receive a delayed intervention later on. b) Not much is said about data analysis except for a presentation of a graph. c) More importantly, the Discussion is rather skimpy. - Previous studies elsewhere which have provided similar findings are not cited.  - There are no limitations to the findings presented. | (a) This is correct. QI collaboratives are a proven approach to improving service delivery – they are not research studies and do not have an experimental design. (b) Data analysis methods are described on pp. 6-7 and results are described on pp. 8-9 (c) We have enhanced the discussion and added limitations and references. |
|  |  |
| Reviewer #3:  The paper describes methods used in scaling up PITC in clear terms. The relevance of the study has been described. The methodologies adequately explained. There may be need to explain further on the one site that did not meet the 95% mark for introducing PITC as this may be a lesson especially for programs that will use the lessons in this for scaling up similar interventions. | All the facilities improved but one of the facilities didn’t achieve 95% due to many factors including workload and change of trained staff. |
| 6. PLOS authors have the option to publish the peer review history of their article ([what does this mean?](https://journals.plos.org/plosone/s/editorial-and-peer-review-process#loc-peer-review-history)). If published, this will include your full peer review and any attached files.   If you choose “no”, your identity will remain anonymous but your review may still be made public.  **Do you want your identity to be public for this peer review?** For information about this choice, including consent withdrawal, please see our [Privacy Policy](https://www.plos.org/privacy-policy). Reviewer #1: Yes: Afolaranmi Tolulope O Reviewer #2: No Reviewer #3: Yes: Dr Patrick Dakum  [NOTE: If reviewer comments were submitted as an attachment file, they will be attached to this email and accessible via the submission site. Please log into your account, locate the manuscript record, and check for the action link "View Attachments". If this link does not appear, there are no attachment files to be viewed.]   While revising your submission, please upload your figure files to the Preflight Analysis and Conversion Engine (PACE) digital diagnostic tool, <https://pacev2.apexcovantage.com/>. PACE helps ensure that figures meet PLOS requirements. To use PACE, you must first register as a user. Registration is free. Then, login and navigate to the UPLOAD tab, where you will find detailed instructions on how to use the tool. If you encounter any issues or have any questions when using PACE, please email us at [figures@plos.org](mailto:figures@plos.org). Please note that Supporting Information files do not need this step. | |
