# Supplementary material for: Improving inpatient provider-initiated HIV testing and counseling in Sierra Leone
Source: PLoS One. 2020 Jul 24;15(7):e0236358. doi: 10.1371/journal.pone.0236358 (PMC7380619; doi:10.1371/journal.pone.0236358)
Supplement: Attachment — Submitted filename: Response to Reviewers.docx [file pone.0236358.s004.docx]

Dear Editor,

We responded to each comments and uploaded the required files.

| **Comments** | **RESPONE** |
| --- | --- |
| 1. Please remove degree after authors' names. | Removed the degrees from the authors’ names in the manuscript |
| 2. Include authors' contributions section. | Authors Contributions included at the end of the manuscript |
| 3. Use the PDSA to illustrate your implementation. | Added a statement on PDSA under the subtitle ‘Project implementation’ |
| 4. A brief description of the study setting in the methods section will be helpful. | Added some statements to describe the study setting under Methods section. |
|  | |
| Submitted the following files |  |
| - A rebuttal letter that responds to each point raised by the academic editor and reviewer(s). This letter should be uploaded as separate file and labeled 'Response to Reviewers'. | √ |
| - A marked-up copy of your manuscript that highlights changes made to the original version. This file should be uploaded as separate file and labeled 'Revised Manuscript with Track Changes'. | √ |
| - An unmarked version of your revised paper without tracked changes. This file should be uploaded as separate file and labeled 'Manuscript'. | √ |

Regards,

Getachew Kassa.
